# Transcription-Dependent Gene Looping of the HIV-1 Provirus Is Dictated by Recognition of Pre-mRNA Processing Signals

**DOI:** 10.1016/j.molcel.2007.11.030

**Published:** 2008-01-18

**Authors:** Kelly J. Perkins, Marina Lusic, Ivonne Mitar, Mauro Giacca, Nick J. Proudfoot

**Affiliations:** 1 Sir William Dunn School of Pathology, South Parks Road, University of Oxford, Oxford OX1 3RE, UK; 2 Laboratory of Molecular Medicine, International Center for Genetic Engineering and Biotechnology, Padriciano, 99-34012 Trieste, Italy

**Keywords:** DNA

## Abstract

HIV-1 provirus, either as a chromosomal integrant or as an episomal plasmid in HeLa cells, forms a transcription-dependent gene loop structure between the 5′LTR promoter and 3′LTR poly(A) signal. Flavopiridol-mediated inhibition of RNA polymerase II elongation blocks 5′ to 3′LTR juxtaposition, indicating that this structure is maintained during transcription. Analysis of mutant or hybrid HIV-1 plasmids demonstrates that replacement of the 5′LTR promoter with CMV or the 3′LTR poly(A) signal with a synthetic element (SPA) permits gene loop formation, suggesting that these interactions are not retroviral specific. In addition, activation of the 5′LTR poly(A) signal or inactivation of the 3′LTR poly(A) signal abolishes gene loop formation. Overall, we demonstrate that both ongoing transcription and pre-mRNA processing are essential for gene loop formation, and predict that these structures represent a defining feature of active gene transcription.

## Introduction

Transcription of protein-encoding genes in eukaryotes is mediated by RNA polymerase II (Pol II) holoenzyme, where drastic alterations to its composition and activity occur during the transcription cycle. Pol II is recruited to promoters at initiation through interaction with multiple transcription factors bound to adjacent or distant regulatory elements (enhancers) by direct enhancer-promoter association, resulting in “looped” conformations that juxtapose otherwise distant genetic elements (West and Fraser, 2005). Initiation structures are replaced by Pol II-associated elongation factors to promote transcription along the gene, generating nascent pre-mRNA. Central to this process is Pol II C-terminal domain (CTD) phosphorylation on serine 5 residues (Ser5) by TFIIH-associated Cdk7 and associated cyclinH (Thomas and Chiang, 2006). Subsequently, elongation factor P-TEFb, comprising the kinase Cdk9 and associated cyclinT, promotes additional Ser2 phosphorylation (Peterlin and Price, 2006). Specific CTD phosphorylation patterns elicit factor recruitment, which modifies underlying chromatin gene structure (Mellor, 2006), promoting cotranscriptional capping, splicing, cleavage, and polyadenylation of the pre-mRNA (Proudfoot et al., 2002).

Many features of this process are defined for HIV-1 (Marcello et al., 2004). The long-terminal repeat (LTR) sequence located at either end of the provirus is divided into three regions, of which U3 contains enhancer and promoter activity, including DNA binding sites for transcription factors USF, NF-κB, and SP1. Full transcription from the 5′LTR requires expression of the virally encoded Tat protein that acts through binding an RNA stem-loop structure called TAR (formed by transcription of the 5′LTR “R” sequence). NF-κB and Tat recruit P-TEFb to TAR and enhance transcriptional elongation (Peterlin and Price, 2006). Finally, R and U5 sequences promote cleavage and polyadenylation of HIV-1 mRNA in the 3′LTR (Bohnlein et al., 1989). Identical sequences in the 5′LTR are blocked from processing activity through interaction with the adjacent major splice donor site (MSD) located 190 nucleotides 3′ to the 5′LTR region (Ashe et al., 1995, 1997).

Several Pol II-transcribed yeast genes adopt a circular conformation associated with transcriptional activation (Ansari and Hampsey, 2005; O'Sullivan et al., 2004). This requires phosphorylated CTD and cleavage/poly(A) factors, which may act as a bridge between the promoter and 3′ end to enhance specificity and/or efficiency of gene transcription. We now demonstrate with chromosome conformation capture (3C) and chromatin immunoprecipitation (ChIP) that integrated HIV-1 provirus induced via cellular (TPA-mediated) or viral (Tat) means shows the existence of looping conformations. Furthermore, inhibiting P-TEFb by flavopiridol (Chao and Price, 2001) severely affects gene loop formation. We also show identical transcription-dependent HIV-1 gene loops form on plasmid DNA by using adapted 3C methodology (plasmid 3C; p3C) in HeLa cells.

Using p3C we show that loop formation was unaffected by replacing either the 5′LTR U3 promoter with the CMV promoter or the 3′LTR with a heterogeneous synthetic poly(A) (SPA) site. Consequently, these interactions are not uniquely a property of retroviruses such as HIV-1. We also show that switching polyadenylation from the 3′LTR to 5′LTR (by MSD mutation) causes a loss of proviral looping. Processing elements involved in 5′-3′ juxtaposition were defined by mutating the endogenous 3′LTR or SPA element, both of which specifically affect looping. Our studies reveal that ongoing transcription and pre-mRNA processing are essential for gene loop formation.

## Results and Discussion

### RNA Pol II Is Asymmetrically Distributed along Transcriptionally Active HIV-1 Provirus

**Figure 1:**
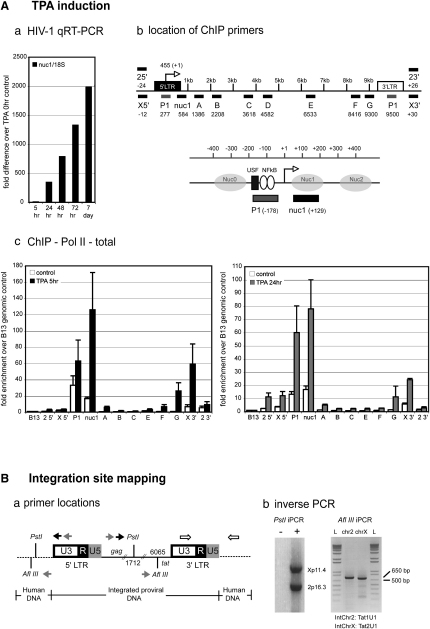
Characterization of Integrated HIV-1 Provirus in U1 Cells (A) (a) HIV-1 mRNA accumulation using qRT-PCR analysis. Values were normalized to 18S rRNA and expressed as a fold induction over untreated U1 cells. (b) Location of ChIP primers relative to the transcription start site (+1) are depicted. Nuc, nucleosomes present on the 5′LTR of latent provirus. Note: P1 does not distinguish between the two LTRs. (c) Pol II qChIP on control (white bars) and TPA-activated U1 cells after 5 (black bars) and 24 hr (gray bars) using the indicated primer and probe sets. Binding was measured relative to B13 (see Experimental Procedures). Error bars represent SEM from n = 3 samples performed in duplicate for each primer set. (B) HIV-1 proviral characterization using iPCR. (a) iPCR primer locations and restriction sites: gray, outer primers; black, nested primers. Open arrows illustrate 3′ HIV-proviral/host cell DNA junctions used to confirm integrate location (see Experimental Procedures). (b) Nested U1 genomic DNA PCR products (+; with Taq polymerase) for integration site mapping and determination of Tat sequences for int-Chr2 (Chr2; Tat1_U1_) and int-ChrX (ChrX; Tat2_U1_).

We used the promonocytic U937 cell line U1 (Folks et al., 1987), which contains two copies of integrated HIV-1 provirus on the short arm of chromosome X and on a rearranged chromosome 6 (Deichmann et al., 1997). U1 cells produce very low levels of HIV-1 mRNA under basal conditions, due to defective viral *trans*-activator Tat mRNAs (Tat1_U1_; mutated initiation codon, Tat2_U1_; H_13_L substitution [Emiliani et al., 1998]). Integrated proviral expression also depends on the surrounding chromatin environment and interaction of the viral Tat *trans*-activator with host factors (Marcello et al., 2004). U1 latent proviral transcription can be markedly induced by exogenous Tat expression (Emiliani et al., 1998) or exposure to the phorbol ester TPA, which induces NF-κB heterodimers to bind within the LTR U3 region (Lusic et al., 2003). Both stimuli cause substantial modification of chromatin structure over the viral 5′LTR promoter (Lusic et al., 2003). Taqman qRT-PCR analysis of U1 RNA after TPA stimulation showed that levels of viral mRNA rose rapidly (400-fold; Figure 1Aa) within 24 hr and further increased up to 7 days.

To analyze proviral transcription in the context of the surrounding chromatin environment, finer mapping was necessary. An inverse PCR strategy was employed (Chun et al., 1997) to map U1 integrates to Xp11.4 and 2p16.3 (Figure 1B); the latter arising from the reciprocal translocation of t(2;6)(p13;p21) (Lee et al., 2002). Integrates are thus referred to as int-ChrX and int-Chr2. Additional iPCR analysis allocated Tat1_U1_ to int-Chr2 and Tat2_U1_ to int-ChrX (Figure 1Bb). Database analysis indicates int-Chr2 and int-ChrX are positioned in transcriptionally silent genomic regions.

U1 HIV-1 proviral Pol II levels were analyzed by quantitative real-time ChIP (qChIP) (Figure 1Ac). Untreated samples showed Pol II occupancy is largely restricted to the 5′LTR, consistent with previous studies (Gomes et al., 2006). TPA treatment showed increased, but asymmetric amounts of Pol II across the HIV-1 provirus, with the strongest signals observed at P1 and adjacent region, nuc1. Distribution was marked by a concurrent increase in bound Pol II within the 3′LTR proximal region (G) and its relative absence from the proviral body, which is maintained at the 24 hr TPA induction time, paralleling sustained provirus transcriptional activity (Figure 1Ac). Induced int-ChrX showed 5-fold higher levels of Pol II occupancy at the 3′ end of the provirus (X3′) than int-Chr2 (23′), indicating that chromatin environment may influence levels of asymmetric Pol II distribution.

### Integrated HIV-1 Provirus Exists in a Looped Chromatin Conformation

In yeast, a similar Pol II profile after transcriptional activation was observed across the *FMP27* gene of *S. cerevisiae* (O'Sullivan et al., 2004), caused by physical interaction between the promoter and 3′ region. We therefore investigated the spatial proximity of proviral regulatory regions under basal or TPA/Tat-induced conditions by using 3C (Dekker et al., 2002). Briefly, 3C defines the proximity between different regions along or between chromosomes by crosslinking chromatin with formaldehyde. Chromatin is then fragmented by a chosen restriction enzyme, diluted to limit random intermolecular interactions, and ligated to covalently join DNA fragments crosslinked to the same complex (intramolecular ligation).

**Figure 2:**
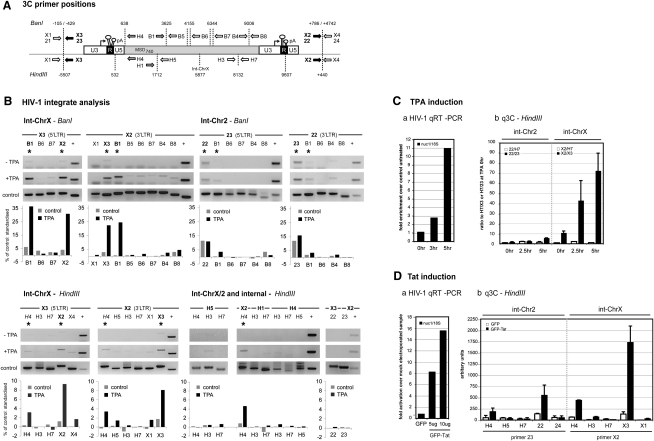
Integrated U1 HIV-1 Proviruses Form Quantitatively Different Looping Conformations (A) Representation of integrated provirus and flanking chromosomal sequence with restriction enzyme sites and primers for BanI and HindIII 3C analysis. Numbers denote distance from 5′ (−) or 3′ (+) proviral ends. Arrows indicate primer direction and name; black/gray arrows refer to primers that detect LTR and the MSD, respectively. HIV-1 long-terminal repeat (LTR) regions (U3, R, and U5), MSD, and polyadenylation sites (pA) are indicated. (B) int-ChrX 3C. Unstimulated (−TPA), cells after 5 hr TPA (+TPA), and control PCR panel (control). Positive lanes (+) signify internal HIV-1 PCR controls on U1 gDNA (control panel) and chromatin (for −/+TPA; see Experimental Procedures). Common PCR primers are shown above the figure, with the second primer shown above each lane. Graphs below represent quantified percentages of 3C product observed compared to PCR control, standardized between − and +TPA samples (using internal PCR controls; see + lane). (C) Quantitative analysis of Tat- or TPA-induced int-ChrX and int-Chr2 loop structures. (a) HIV-1 qRT-PCR at 0, 3, and 5 hr post-TPA treatment with nuc1 primers (Figure 1A) standardized to 18S rRNA transcription. (b) q3C HindIII-digested U1 chromatin analysis; primers used to detect the “loop”: interaction (primers 22/23 for int-Chr2 and X2/X3 for int-ChrX) compared to the adjacent amplified fragment (primers 22/H7 or X2/H7). (D) (a) HIV-1 qRT-PCR treated with 0 (10 μg GFP control), 5, or 10 μg of GFP-tagged Tat protein. (b) q3C HindIII analysis of U1 chromatin treated with 10 μg Tat-GFP or GFP. Error bars represent SEM from n = 6 samples from two separate chromatin preparations (except 22/23 and X2/X3 in Tat induction analysis where n = 9, from three separate chromatin preparations).

We used int-ChrX or int-Chr2 flanking primers with internal proviral primers (Figure 2A) to discriminate between integrates. Chromatin preparations were digested with BanI or HindIII restriction enzymes from noninduced or TPA-treated cells, which increase transcript levels 11-fold (Figure 2Ca). Representative gels of 3C PCR products obtained under noninduced and stimulated conditions are shown in Figure 2B, where positive control lanes show expected PCR products from the intramolecular ligation of BanI- or HindIII-digested fragments and graphs indicate percentage products compared to the PCR control. Upon TPA induction, a specific increase in 3C products was apparent for int-ChrX with BanI- and HindIII-digested chromatin. With BanI, stronger 3C products were observed, specifying the close spatial proximity of the 5′LTR with the 3′LTR (using primers X3/X2) and both LTRs with the adjacent fragment to the 5′LTR containing the MSD (primers X3/B1 and X2/B1 respectively). All 3C products were defined by DNA sequencing. Untreated cells showed low levels of the same products, consistent with the presence of low, albeit detectable, full-length HIV-1 mRNAs under basal conditions (Jeang et al., 1993).

HindIII int-ChrX analysis (Figure 2B) gave a similar profile to BanI: 3C PCR products were detected with primers that detect interactions between the 5′LTR and 3′LTR (X3/X2) and with the adjacent fragment containing the MSD (X3/H4 and X2/H4). As a HindIII site is positioned between the U3/R and U5 region (Figure 2A), both the LTR-LTR interaction and 5′LTR U3 and R region with its adjacent fragment containing the U5 and MSD could be more precisely defined (primers H4/X3). As shown in Figure 2B, the principal 3C product amplified was obtained with the primers X3/X2 and indicates an interaction between the 5′LTR U3 and R region with the U5 region of the 3′LTR.

To determine whether interactions defined by 3C were restricted to the LTRs and their flanking regions or if internal regions of the HIV-1 provirus interact with each other, we performed intraproviral analysis. We also performed interchromosomal 3C analysis to investigate whether the two integrates interact (Figure 2B). Neither analysis resulted in significantly detectable products, demonstrating that loop formation appears to be both discrete to each integrate and largely restricted to fragments encompassing duplicated LTRs and short flanking chromosomal regions.

### int-ChrX and int-Chr2 Proviruses Show Quantitative Differences in Gene Loop Formation

ChIP data show that, in contrast to int-ChrX, Pol II signals adjacent to the 3′LTR of int-Chr2 remain at basal levels after TPA stimulation, suggesting that gene loop formation is less pronounced (Figure 1Ac). We analyzed int-Chr2-specific 3C products with BanI-digested chromatin (Figure 2B). 3C interactions were detectable between the LTRs but did not change significantly upon TPA stimulation. We did, however, observe some increase in the 3C interaction between both LTRs and the MSD (primers 23/B1 and 22/B1). To compare relative levels of gene loop formation between integrates after transcriptional activation, we employed quantitative real-time 3C (q3C) analysis of HindIII-digested chromatin from 5 hr TPA-treated U1 cells. As shown in Figure 2Cb, a modest increase in the 5′-3′LTR 3C product from int-Chr2 was observed over basal levels after activation, whereas int-ChrX showed a significantly higher increase. The difference between integrates was more striking when promoter-terminator interactions were compared to amplification of an adjacent fragment by using 22 (int-Chr2) and X2 (int-ChrX) with H7 (3.7-fold for int-Chr2 and 63-fold for int-ChrX).

### Tat-Mediated HIV-1 Transcriptional Activation Also Stimulates Loop Formation

HIV-1 transcription is enhanced by the viral Tat *trans*-activator through interaction with the 5′LTR TAR transcript (Peterlin and Price, 2006). Tat-associated TAR is known to augment transcriptional initiation and elongation of Pol II. These are mediated by Tat interaction with nuclear proteins possessing chromatin remodeling activity and cellular kinases (most notably, P-TEFb), which phosphorylate the Pol II CTD (Marcello et al., 2004).

We thus compared the effect of Tat- versus TPA-mediated induction on HIV-1 gene loop formation. As both integrates have a defective *tat* sequence (Figure 1B), Tat was supplied in *trans* by electroporation of a Tat-GFP expression vector. GFP-Tat *trans*-activation resulted in a 16-fold stimulation of HIV-1 mRNA levels (Figure 2Da). We undertook HindIII q3C analysis and observed Tat-dependent 3C products, indicating gene loop formation over both proviral integrates (Figure 2Db). Weaker LTR-MSD interactions were also detected (primers 23/H4 and X2/H4). Again, int-ChrX gave a much higher response than int-Chr2 to exogenous Tat (12-fold as compared to 4-fold). This result parallels TPA induction and suggests HIV-1 gene loop formation is a feature of transcriptional initiation and elongation.

Results obtained from 3C analysis thus demonstrate the existence of two transcription-dependent proviral conformations. First, a promoter-poly(A) signal interaction occurs across the provirus, signifying that loop formation is separate to the site of transcriptional termination, which occurs at least 1 kb downstream of both integrate 3′LTRs as determined by RT-PCR (data not shown). A second interaction is observed between both LTRs and a proviral sequence adjacent to the 5′LTR containing the MSD. These results are summarized as a ribbon diagram (Figure 7A).

### HIV-1 Gene Loops Are Detected by Plasmid 3C Analysis of Transfected HIV-1 Provirus

**Figure 3:**
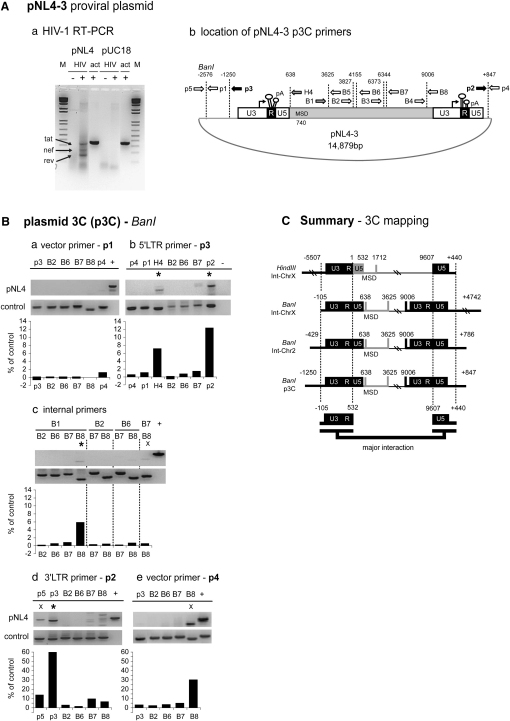
p3C Analysis Indicates HIV-1 Loop Formation Occurs on a Plasmid Template (A) (a) RT-PCR analysis of pNL4-3-transfected HeLa cell RNA detecting HIV-1-specific transcripts. HIV, HIV-1 primers; act, actin control primers; M, DNA marker; pUC18, vector backbone, − or + RT. (b) p3C BanI restriction sites and primer position and direction are numbered as in Figure 2A; black and gray arrows denote primers corresponding to LTR and MSD regions, respectively; and plasmid-specific primers are allocated according to the BanI position from the 5′ (−) 3′ (+) proviral ends. (B) BanI p3C analysis shows LTR-LTR and LTR/MSD juxtaposition. Positive PCR panels (control) were used to demonstrate that primer sets amplify correct products. + lane signifies internal HIV-1 PCR controls; common PCR primers are shown above the figure and second primers above each lane. Significant 3C products (^∗^) and those derived from adjacent fragment ligation (X) are denoted. Graphs below represent quantified percentage of 3C product observed compared to PCR control, as per Figure 2B (note: chromatin preparations for p2 (d) and p4 (e) were concentrated as outlined in the Experimental Procedures). (C) 3C and p3C summary. HIV-1 provirus with restriction sites present in pNL4-3, int-ChrX, int-Chr2, and their flanking regions are as indicated. Major LTR-LTR interactions are shown in black; regions showing partial interaction are in gray. Lower brackets define minimal interacting regions from combined 3C and p3C data.

We initially considered that HIV-1 proviral gene loops may depend on their chromatin environment. However, difficulty in manipulating specific sequences in a genomic context encouraged us to determine if looping could be demonstrated on a transfected template. Plasmids have been shown to adopt nuclear plasmid transcription domains separate from chromosomal territories (Binnie et al., 2006). We transfected the proviral plasmid pNL4-3 (Adachi et al., 1986) into HeLa cells and showed that HIV-1-specific transcripts were detectible by RT-PCR analysis after 48 hr (Figure 3Aa).

To detect specific pNL4-3 loop products, we adapted 3C into a plasmid 3C technique (p3C), which requires additional purification steps by repeated centrifugation through sucrose cushions necessary to remove excess transfected cytoplasmic plasmid accumulated around the nuclear periphery. We performed BanI p3C with internal HIV-1 primers previously used for chromosomal 3C analysis (Figure 2A) with pUC18 vector primers p1–p5 (Figure 3Ab). Identical p3C products were detected as seen with transcriptionally activated HIV-1 chromosomal integrants (Figure 3B). Thus, primer p3 (5′LTR) amplified specific 3C products with primers H4 and p2, showing the same MSD and 3′LTR interaction. Other primer combinations confirmed these findings and were subsequently reproduced with quantitative real-time p3C analysis (q-p3C; see Figure 6). Although adjacent BanI fragments did occasionally amplify 3C products (denoted by x; Figure 3B), most primer pairs unrelated to MSD or LTR interactions gave only background 3C levels. These negative controls are striking, as plasmids transcribing homologous sequences colocalize into plasmid transcription domains (Binnie et al., 2006). As specific gene loop products were detectable between proviral LTR and LTR-MSD regions, we suggest that this phenomenon is occurring in *cis*. These results are consistent with the lack of intrachromosomal 3C signal between int-ChrX and int-Chr2 in U1 cells (Figure 2B). Combined 3C and p3C analyses show that major interactions occur between the 5′LTR U3 promoter and 3′LTR U5 poly(A) signal as well as between each LTR and the 5′ positioned MSD signal (Figures 3C and 7B).

### Loop Formation Is Reflected in Pol II CTD Phospho-Ser2 and -Ser5 Distribution

**Figure 4:**
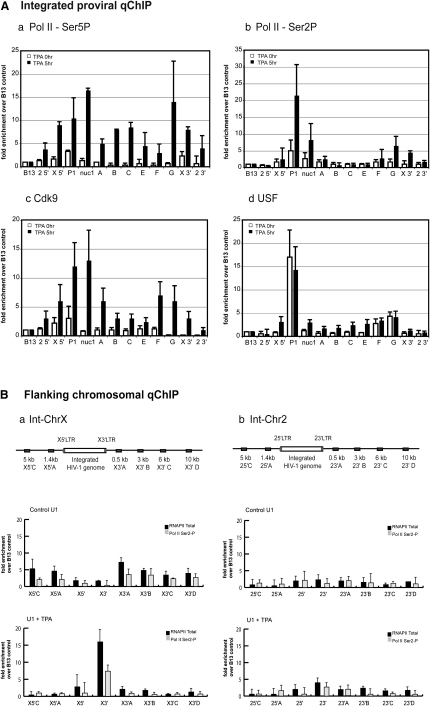
Occupancy of Pol II and Associated Factors across Transcriptionally Active Provirus and Flanking Chromosomal Regions (A) Asymmetric distribution of Pol II Ser5P (a) Ser2P (b), CDK9 (c), and USF (d) association was determined by qChIP assay in control (white bars) and 5 hr TPA-activated U1 cells (black bars). Immunoprecipitated DNA was analyzed by real-time PCR using the primer sets as described in Figure 1. ChIP signal levels are not comparable between antibodies. Note: P1 does not distinguish between the two LTRs. (B) Flanking chromosomal analysis of proviral integrates indicates differential response to TPA stimulation for (a) int-ChrX and (b) int-Chr2. Error bars represent SEM from n = 3 samples performed in duplicate for each primer set.

Pol II CTD phosphorylation changes through the transcription cycle and is involved in several postinitiation steps (Meinhart et al., 2005). We therefore tested whether loop formation correlates with Pol II CTD phosphorylation and thus with polymerase processivity. qChIP analysis with antibodies recognizing phospho-Ser5 (Ser5P) showed barely detectable levels at the promoter and absence from the proviral body for noninduced U1 cells (Figure 4Aa). Upon activation, Ser5P was present throughout the provirus with the highest occupancy (almost 2-fold higher) at the 5′LTR and 3′LTR regions. Consistent with high levels of viral transcription (Figure 1A), high Ser5 levels persisted for longer time periods (24 hr TPA induction; Figure S1 available online).

Upon transcriptional activation, very low amounts of Ser2 were detected within the provirus (Figure 4Ab), with a higher, asymmetric distribution pattern in particular over the 5′ and 3′LTRs (nuc1 and G, respectively). The highest signal was detected by P1 primers in both 5′ and 3′LTR regions. In agreement with studies on HIV-1 LTR reporter genes (Bres et al., 2005), higher Ser2P levels were detected in the proviral body during longer, 24 hr TPA induction times (Figure S1). The observation that Ser2P/Ser5P is more easily detected than total RNAP II in the proviral body after TPA induction (Figure 1Ac) is consistent with studies on other cellular genes (Gomes et al., 2006). Asymmetric distribution of Ser2P/Ser5P also mirrors 3C data showing that 5′ and 3′LTRs are juxtaposed. Moreover, qChIP data are consistent with our observation that the integrates differ in their response to transcriptional stimuli as stronger Ser2P/Ser5P ChIP signals are observed at the earlier TPA induction time for the int-ChrX provirus (Figures 4Aa and 4Ab). Together these data indicate that int-ChrX displays higher transcriptional inducibility and activity.

To confirm Ser2P/Ser5P profiles, anti-CDK9 qChIP detected small amounts of CDK9 in the 5′LTR under basal conditions **(**Figure 4Ac), consistent with studies that show CDK9 at promoter regions of transcriptionally inactive human genes (Gomes et al., 2006). TPA induction (5 hr) causes CDK9 accumulation at both ends of the provirus with a more even distribution across the provirus at 24 hr (Figure S1). Consequently, CDK9 distribution patterns mirror those of Ser2P. As H_13_L-substituted Tat (int-ChrX) retains some of its *trans*-activating activity, TPA-induced proviral expression results in production of endogenous Tat protein in U1 cells, effectively making these cells a complete HIV-1 transcription environment. We predict that Tat activity shifts CDK9 phosphorylation from Ser2P to Ser2P/Ser5P, explaining the marked similarity of their ChIP profiles with CDK9 (Figures 4Aa–4Ac). Finally, we analyzed upstream stimulatory factor (USF), which binds the HIV-1 LTR U3 region (Figure 1Ab) irrespective of the transcriptional state of the provirus (Giacca et al., 1992). Under both noninduced and TPA-activated conditions, USF was strongly detected at the 5′LTR and more weakly at the 3′LTR of the viral genome, consistent with a model in which for the uninduced state, hypophosphorylated Pol II binds the 5′LTR region (Figure 4Ad).

As proviral expression is dependent on the surrounding chromatin environment (Marcello et al., 2004), we analyzed the flanking 16 kb region of both integrates with qChIP (Figure 4B). Although there was little change in the int-Chr2 profile (Figure 4Bb), int-ChrX showed significant increases in both Pol II and Ser2P occupancy at the 3′LTR upon 5 hr TPA treatment. Thus, differences in loop formation may arise from flanking regions influencing the ability of integrated provirus to respond to transcriptional stimuli.

### HIV-1 Long-Terminal Repeat Juxtaposition Is Dependent on Ongoing Transcription

**Figure 5:**
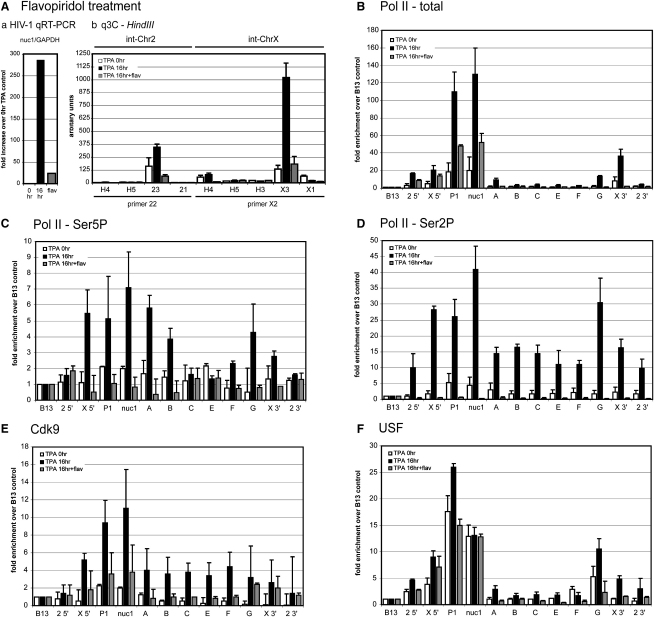
Flavopiridol Inactivates HIV-1 Transcription by Blocking CTD Ser2P and Ser5p, Leading to Loss of Gene Loop Structure (A) (a) HIV-1 mRNA levels (qRT-PCR) and (b) q3C (see Figure 2) prior to (white bar) or after 16 hr TPA treatment (black bar) followed by 5 hr of flavopiridol treatment (500 nM; gray bar). (B–F) qChIP analysis for total RNAP II, Ser5P, Ser2P, CDK9, and USF respectively was performed for control (white bars), transcriptionally active (TPA induced; black bars), and transcriptionally blocked (flavopiridol treatment after TPA induction) U1 cells (gray bars). ChIP signal levels are not comparable between antibodies. P1 primers amplified from both 5′ and 3′LTR DNA. Error bars represent SEM from n = 3 samples performed in duplicate for each primer set, except primers 22/23 and X2/X3 (n = 12), which are from four separate chromatin preparations.

To strengthen our observations that gene loop structures exist only when HIV-1 provirus is transcriptionally active, we used the drug flavopiridol, which specifically represses CDK9 kinase activity, resulting in its inability to phosphorylate the Pol II CTD (Chao and Price, 2001). HIV-1 transcription was induced by 16 hr TPA treatment and then blocked by further treatment with flavopiridol for 5 hr (Figure 5Aa). We performed q3C, focusing on formation of int-Chr2 and int-ChrX proviral gene loops. Consistent with the original 3C analysis (Figure 2C), int-Chr2 provirus forms a weakly inducible gene loop after 16 hr TPA induction (primers 22 and 23), whereas int-ChrX forms a strongly inducible gene loop (primers X2 and X3). Dramatically, TPA-stimulated cells treated with flavopiridol for 5 hr caused 3C signal levels to drop to control (unstimulated) levels for both proviruses, suggesting that ongoing transcriptional elongation is indeed required for gene loop maintenance. This was strengthened by additional studies on chromatin from cells subjected to flavopiridol treatment in the absence of TPA induction in which 3C products corresponding to low levels of HIV-1 looping in untreated U1 (control) cells were significantly reduced (Figure S2).

To verify that transcriptional inhibition correlates with blocking CDK9 kinase activity, we performed a full set of qChIP experiments under these conditions. Flavopiridol treatment caused a predicted loss of CDK9 across the proviral genome and Ser2P/Ser5P from the integrated provirus, supporting the observation that CDK9 acts on CTD Ser2 and Ser5 in transcriptionally activated U1 cells (Figures 5C–5E). Significantly, HIV-1 proviral distribution pattern for total Pol II and USF was differentially affected by flavopiridol treatment (Figures 5B and 5F). Instead of reducing all signals to low levels, Pol II and USF signals were selectively lost from the proviral body and 3′ flank but were maintained over both LTR regions (P1) and immediately downstream from the 5′LTR transcription start site (nuc1). These data therefore support the view that gene loop formation is consequent to formation of the USF-dependent Pol II initiation complex.

### HIV-1 Gene Loop Formation Is Dependent on Competing 5′ and 3′LTR Poly(A) Site Recognition

**Figure 6:**
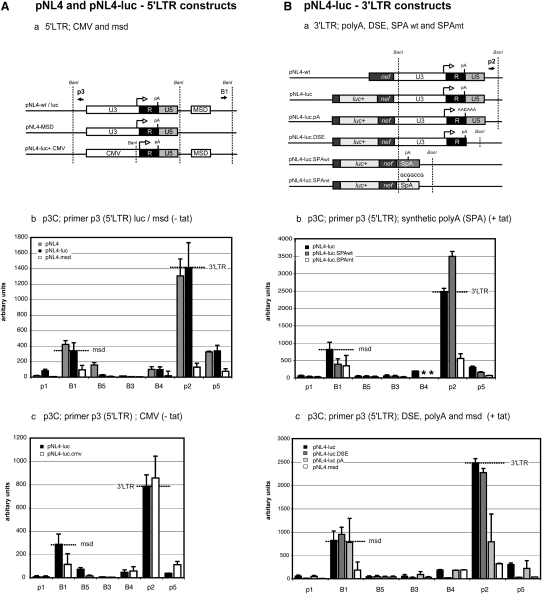
Functional MSD and 3′LTR Poly(A) Signals Are Required for Transcription-Dependent Loop Formation of pNL4 HIV-1 Provirus (A) (a) pNL4 5′ end mutant constructs used in p3C analysis showing primers used to detect HIV-1 LTR or CMV promoter (p3) and the major splice donor (MSD; B1). Primer numbering and positions, poly(A), LTR, and MSD regions are as in Figure 3. Arrows indicate transcription start sites. (b) Real-time q-p3C analysis of comparative loop formation using primer p3 (5′LTR) using pNL4, derivatives pNL4-luc, and pNL4.msd and (c) pNL4-luc compared to the CMV promoter construct pNL4-luc.CMV. (B) (a) pNL4-luc 3′ end poly(A) and SPA constructs used in p3C analysis; primer numbering and positions are as in Figure 3, with luciferase (luc+) reporter gene, poly(A), and LTR regions as shown. Real-time q-p3C analysis of comparative loop formation using primer p3 (5′LTR) in analysis of the SPA wild-type and mutant (b) HIV-1 poly(A) and DSE mutants (c). Asterisks denote absence of the B4 primer binding site for SPA constructs. For all graphs, dotted lines show values of pNL4-luc obtained for 5′LTR/MSD and 5′LTR/3′ end (3′LTR) products with (+) or without (−) cotransfected Tat expression vector. Primer p3 was used in combination with the primers depicted below all graphs. Error bars represent SEM between values from separate chromatin preparations performed in triplicate (n = 6), except for p3/B1 (n = 12) and p3/p2 (n = 15).

**Figure 7:**
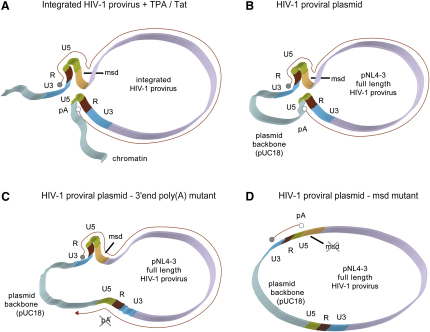
Ribbon Diagrams Illustrating HIV-1 Interactions as Determined by 3C Analysis (A) Potential interactions in the HIV-1 proviral sequence upon TPA or Tat stimulation for int-ChrX. Also illustrated is the hypothesized conformation of pNL4-3 in (B) wild-type (C) in the absence of a functional 3′ end poly(A) signal (as evidenced by the HIV-1 poly[A] and SPA mutants) and (D) without a functional MSD. Dashed lines represent transcription (with gray and white circles denoting promoter/poly[A] sites respectively), with LTRs, MSD, and poly(A) hexamer as indicated.

With the development of the plasmid 3C technique (Figure 3), we directly manipulated HIV-1 sequences required for gene loop formation. We analyzed a noninfectious pNL4 derivative (pNL4-3.R-E-; henceforth called pNL4-luc) and the effect of activating the 5′LTR poly(A) signal by inactivating the MSD. Normally, the MSD suppresses the 5′LTR poly(A) signal through the negative interaction of U1snRNP 70 kDa subunit (Ashe et al., 2000; Figure S3). Furthermore, U1snRNP recognition requires definition of a complete intron transcript, including 3′ splicing signals (Ashe et al., 1995). We mutated the pNL4 MSD (Figure S3A), confirmed that the 5′LTR poly(A) signal was used by RNase protection analysis (RPA; Figure S3B), and performed BanI q-p3C analysis (Figure 6Ab). Dramatically, MSD point mutation significantly affected both gene loop conformations. In effect, the pNL4 plasmid loses its contorted structure associated with the two gene loops and adopts a more open circular conformation. This structural switch correlates with a switch in transcript length from full-length HIV-1 transcripts utilizing the 3′LTR poly(A) signal to a short transcript that utilizes the 5′LTR poly(A) signal (Figure 7D). As pNL4-luc gave comparable results to those observed for pNL4 (Figure 6Ab), it was used as a template for further mutational analysis.

### HIV-1 Gene Loop Formation Is LTR Independent but Requires a Functional 3′ Poly(A) Signal

We wished to determine whether 5′-3′ gene looping was unique to retroviruses such as HIV-1, which differ from normal transcription units in being flanked by identical LTR sequences. We therefore replaced the 5′ HIV-1 U3 promoter with a CMV promoter (Figure 6Aa), while maintaining the 3′LTR poly(A) site. 5′-3′ juxtaposition was unaffected (1.08-fold increase compared to wild-type pNL4-luc; Figure 6Ac). However, a 2.5-fold drop in 5′LTR/MSD product was observed, which may arise from the lack of the 5′ R/U5 region amplified by the 5′LTR p3 primer. R contains the TAR element, which is a known influence in efficiency of 5′ poly(A) site suppression (Furger et al., 2001).

As mutating the MSD directly influences transcription by poly(A) site usage, the ability of Pol II to reach the 3′LTR may be affected. Loss of loop products could therefore be linked to the lack of an intact RNA extending between the 5′ and 3′LTR. We therefore analyzed a number of 3′ end constructs and their effect on loop formation, while maintaining the 5′LTR (Figure 6B). Using RPA analysis, we determined that these constructs did not utilize the 5′ poly(A) site and are expected to produce transcripts that reach the 3′LTR (data not shown). We cotransfected a Tat expression vector to prevent any alteration in transcription rates of the mutants caused by potential defective processing of Tat mRNA. Two 3′LTR poly(A) signal mutants were analyzed (Figure 6Bb): one inactivating the essential hexamer sequence (AAUAAA to AACAAA; pNL4-luc.pA) and the other removing the U5 region containing the GU-rich DSE element (pNL4-luc.DSE). Mutation of the 3′ poly(A) hexamer resulted in a 3-fold decrease in 5′-3′ LTR p3C product while maintaining wild-type values between the 5′LTR-MSD (0.95-fold; Figure 6Bc). This shows poly(A) site recognition is essential for juxtaposition of the 5′-3′ region. Interestingly pNL4-luc.DSE showed little change (0.9-fold for both the 5′-3′LTR and 5′LTR-MSD compared to pNL4-luc), which may be explained by the presence of a redundant GU-rich sequence brought in close proximity to the hexamer after DSE deletion (Figure S5i). We extended this analysis by replacing the 3′LTR with a heterologous strong synthetic poly(A) (SPA) site (Levitt et al., 1989) while maintaining the 5′LTR (Figures 6Ba and 6Bb). Interestingly, p3C analysis showed that replacing the entire 3′LTR region with SPA is not detrimental to 5′-3′ juxtaposition (1.4-fold increase). Conversely, the nonfunctional SPA site (pNL4-luc.SPAmt) abolishes this interaction more effectively than the endogenous poly(A) mutant (4.5-fold decrease). However, it may be possible that the presence of a weaker AAUAAA signal within the U3 region of the 3′LTR (Figure S5ii) could partially compensate as a cryptic poly(A) site in the pNL4-luc.pA mutant. In addition, values obtained for B1/p3 products may be affected by the detection of circularized partial digestion products. We therefore performed p3C analysis with a primer facing in the same direction as p3 (H4.1) for all constructs (Figure S4) and obtained comparable values. Overall results from p3C analysis allow us to conclude that HIV-1 gene loop formation requires coupled pre-mRNA processing.

### Conclusions

We have demonstrated that a specific gene loop conformation is imposed on integrated HIV-1 provirus after transcriptional activation and that different chromosomal contexts of these two integrates modulates both transcriptional levels and the consequent capacity to form gene loop structures. U1 int-Chr2 provirus is only weakly inducible by TPA or Tat and forms lower levels of gene looping that only increase a few fold after induction (Figures 2C and 2D). This correlates with relatively low levels of transcription apparatus associated with this provirus before or after TPA induction (based on ChIP analysis, Figures 1A and 4A). Instead, int-ChrX provirus is strongly activated by TPA showing higher levels of active Pol II, CDK9, and USF recruitment and a commensurate high level of gene loop conformation (Figure 2). Another conformational change observed for both proviral genomes was detected between their LTRs and the MSD region adjacent to the 5′LTR (Figure 2B), presumably reflecting the functional interplay between the MSD and 5′LTR poly(A) signal (Ashe et al., 1995, 1997). These data have also been extended and confirmed by p3C analysis (Figures 3 and 6). That we observe specific LTR-LTR and LTR-MSD interactions argues that these gene conformations are not dictated by specific chromatin structures but rather are permissively formed across the proviral genome as long as there is ongoing transcription. Strikingly, p3C analysis demonstrates that gene loop formation is entirely dependent on interplay between pre-mRNA splicing and alternative polyadenylation, as exemplified by retroviruses such as HIV-1. As inactivation of the MSD causes a significant drop in both gene loops; these structures appear to not only depend on ongoing transcription but also act to define the correct RNA processing pattern to allow effective HIV-1 gene expression. This is confirmed by direct mutation of the 3′LTR poly(A) hexamer, which also causes a significant loss of LTR-LTR juxtaposition. Furthermore, either the 5′LTR promoter or 3′LTR poly(A) signal can be functionally replaced with heterologous elements and still maintain full gene loop levels.

Applying 3C analysis to investigate long-range associations between noncontiguous chromosomal segments has become widespread. Clustering of coregulated genes has been reported (Simonis et al., 2006; Zhao et al., 2006), suggesting the existence of so-called transcription “factories” (Pombo et al., 2000). Also, substantial distances between gene regulatory sequences (enhancers) and target promoters can be explained by their 3D juxtaposition detected by 3C analysis (Fraser, 2006; Splinter et al., 2004; Vakoc et al., 2005). For example, the interleukin gene cluster in mouse has specific chromosomal structures called SATBs, required for gene expression, and looping with their adjacent genes promotes effective gene expression (Cai et al., 2006). However, our study reports a different type of gene conformation, reflecting the dynamic structural changes associated with the actual transcription process. We first reported this phenomenon for *S. cerevisiae* (O'Sullivan et al., 2004) where *FMP27* gene loop formation was shown to be dependent on Pol II CTD Ser5P and kinase Kin28p. It also appears that that specific poly(A) factors (Ssu72p and Pta1) are required for loop formation (Ansari and Hampsey, 2005). Furthermore, recent results also imply a role for TFIIB in gene loop formation (Singh and Hampsey, 2007). Finally, in the human mitochondrial episomal genome, a promoter-terminator loop juxtaposition on the heavy strand depends on the transcription factor mTERF, required for rRNA transcription initiation and termination (Martin et al., 2005). Our studies extend this gene loop phenomenon to higher eukaryotes, to an integrated viral genome, and on a plasmid template. The generality of transcription-dependent gene loops in eukaryotes is therefore predicted.

Whether gene loop structures are a cause or a consequence of the transcription process has yet to be determined. However, additional insights derive from this study. First, potent inhibition of gene loop formation by flavopiridol treatment, which blocks transcription elongation, but not formation, of the Pol II preinitiation complex, is important. This is judged by the continued presence of USF and Pol II on the HIV-1 5′LTR promoter even after loop destruction. These data argue, at least for HIV-1 transcription, that the early elongation phase is essential for gene loop formation. Second, these observations indicate that loop structure appears unstable, requiring ongoing transcription for maintenance. Third, we have discovered a direct connection between pre-mRNA processing and gene loop formation. Clearly, recognition of the poly(A) signal is required for gene loop formation, and this is itself dependent on coupled splice site recognition. We thus propose that gene loop structures constitute an integral part of defining a successful vertebrate gene transcription unit.

## Experimental Procedures

### HIV-1 Inverse PCR Analysis

Chromosomal regions flanking U1-(U937) cell line proviral integrates were defined according to Chun et al. (1997). U1 genomic DNA (2 μg) was digested with PstI (New England Biolabs) for 5 hr at 37°C, with 200 ng ligated overnight at 4°C. Ten nanograms of DNA was used in a PCR with primers across the viral genome and host cell DNA, then a nested reaction was performed with HIV-1 primers (Han et al., 2004). Tat status was determined with AflIII-digested DNA by using a primer 5′ to the Tat first exon with int-ChrX/2 primers proximal to HIV-1 5′LTR chromosomal AflIII sites. 3′ flanking regions were defined by using a HIV-1 primer spanning nef/U3 (nt9091-9197) and primers specific to int-ChrX/2. All PCR products were fully sequenced. Primer sequences and locations are available in the Supplemental Data.

### Plasmid Constructs

Recombinant HIV-1 pNL4-3 plasmid (Adachi et al., 1986) used in p3C/integrated into the U1-(U937) monocytic cell line (U1) and the MSD-positive vector control (Ashe et al., 1995) have been previously described. All pNL4-3 and pNL4-3.R-E- derivatives were created as described in the Supplemental Data. RT-PCR analysis on transfected HeLa RNA used primers and conditions as described (Cannon et al., 1994).

### Tissue Culture

U1 and HeLa cells were maintained at low-passage level in RPMI 1640 (+20 mM l-glutamine, 10% FCS). U1 cells (density 5 × 10^5^) were treated with 10^−7^M TPA (Sigma) for the times indicated. HeLa cells (2 × 10^7^) were transfected with 5 μg pNL4-3/pNL4-3.R-E- proviral constructs or pUC18 control using Fugene (Roche). 0.5 μg Tat expression vector (Ashe et al., 1997) was cotransfected for pNL4-3.R-E- 3′ mutant studies. Tat-GFP/GFP control plasmids (Lusic et al., 2003) were electroporated into U1 (1 × 10^7^) cells (Gene Pulser, Biorad) with transfection efficiency measured by FACS analysis. At time points indicated, cells were washed twice with 1×PBS for RNA purification or resuspended in fresh medium and crosslinked for ChIP and 3C analysis. Flavopiridol-treated samples were obtained by treating cells with TPA (1 × 10^−7^M) for 16 hr and then adding 500 nM flavopiridol for 5 hr (Division of AIDS, NIAID, NIH). HIV-1 transcript levels were measured with TaqMan (qRT-PCR) using HIV-1 nuc1 primers and housekeeping gene controls (GAPDH/18SrRNA) on an AbiPrism 7000 machine (Applied Biosystems).

### ChIP

ChIP was performed as described (Lusic et al., 2003). To detect chromosomal flanking regions, pellets were sonicated (Bioruptor sonicator) to obtain fragments of 1–1.5 kb. ChIP antibodies are as follows: Pol II (H224), USF (C20), and CDK9 (H-169) (Santa Cruz); Pol II Ser2P (H5) and Ser5P (H14) (Covance). Diluted input concentrations (1/10 and 1/100) were used as standards for each primer and TaqMan probe set. Real-time PCR used TaqMan technology (AbiPrism 7000; Applied Biosystems), standardized to primer efficiency. Fold antibody enrichment (occupancy) was calculated as a percent of input material, normalized to an unrelated genomic region (B13; Lusic et al., 2003). Primer sequences are available on request.

### 3C and p3C

3C (Tolhuis et al., 2002) was performed on 5 × 10^7^ U1 cells with modifications (Vakoc et al., 2005). For p3C analysis, HeLa cells (2 × 10^7^) were crosslinked as described (Tolhuis et al., 2002). Nuclei were isolated by centrifuging for 5 min at 13,000 rpm were resuspended in 500 μl lysis buffer (10 mM Tris [pH 8.0], 10 mM NaCl, and 0.2% NP-40), underlayed with lysis buffer + 24% sucrose and centrifuged for 5 min at 13,000 rpm. This step was repeated twice, once with 20% sucrose, and then nuclei were resuspended in 750 μl 1× restriction buffer. Fifty microliters of nuclei was digested with 800 U of BanI or HindIII (NEB) at 37°C overnight, with subsequent steps as previously described (Vakoc et al., 2005), except p3C products were column purified (QIAGEN) instead of precipitated. 3C and p3C templates were resuspended/eluted in a total volume of 200/100 μl TE, respectively. Note: p3C analysis using primers p2 and p4 (Figures 3Bd and 3Be) used 100 μl nuclei in 50 μl elution volume. Two microliters of input was used in a standardized PCR reaction of 32 cycles of 95°C for 45 s, 58°C for 45 s, 72°C for 1 min, and final extension for 5 min at 72°C. HIV-1 internal PCR controls were obtained by PCR amplification using 1 μg U1 genomic DNA/pNL4-3 as above, column purified, and quantified to supply a random pool of religated products. Equimolar amounts of PCR products were digested 5 hr at 37°C and ligated overnight at 4°C, and a 1/500 dilution was used for agarose gel analysis. Real-time (qRT-PCR) 3C used 2 μl of input in triplicate (Corbett Rotor-Gene 3000 with QuantiTect SYBR Green [QIAGEN)). Agarose PCR products were quantified with ImageQuant IQ Tools Version 2.2 (Amersham). qRT-PCR products were analyzed with Rotor-Gene6 software and standardized to (1) relative primer efficiency and (2) values from internal HIV-1 PCR products (primers flanking the nt3625 BanI site for HindIII and nt 3525–3760 for BanI analysis). 3C/p3C primer details are available on request.

## Supplemental Data

Document S1. Supplemental Experimental Procedures and Five Figures
