# Supplementary material for: Transcription-Dependent Gene Looping of the HIV-1 Provirus Is Dictated by Recognition of Pre-mRNA Processing Signals
Source: Mol Cell. 2008 Jan 18;29(1):56–68. doi: 10.1016/j.molcel.2007.11.030 (PMC2225447; doi:10.1016/j.molcel.2007.11.030)
Supplement: Document S1. Supplemental Experimental Procedures and Five Figures [file mmc1.pdf]

Molecular Cell, Volume *29*

Supplemental Data

Transcription-Dependent Gene Looping of the HIV-1 Provirus Is Dictated by Recognition of Pre-mRNA Processing Signals

Kelly J. Perkins, Marina Lusic, Ivonne Mitar, Mauro Giacca, and Nick J. Proudfoot

SUPPLEMENTAL EXPERIMENTAL PROCEDURES

A. Inverse PCR (iPCR):

BAC numbering is according to NCBI Mapviewer annotation; http://www.ncbi.nlm.nih.gov/mapview/

i) primer location and restriction sites for tat genomic sequence analysis

Int-ChrX:	*Afl*III nt16022	primer nt16381;	5′-GAGGTAAAGAATGTCATAGCTTGTGGGC-3′
Int-Chr2:	*Afl*III nt21827	primer nt19680;	5′-CCTGTGGAACTCTGAGCCAATAAACC-3′
HIV-1:	*Afl*III nt6065	primer nt5600;	5′-GAGTGAAGCTGTTAGACATTTTCCTAGGATATGGCTC-3′

ii) integration site confirmation for int-ChrX and int-Chr2 (BAC position as reference)

Int-ChrX:	primer ChrXR	nt15738-5768	5′-CAGAACTCTTCCAGCTGCTATTCAGTGCTTG-3′
Int-Chr2:	primer Chr2R	nt22013-21991	5′-CCTGTGGAACTCTGAGCCAATAAACC-3′
HIV-1:	primer nef/U3	nt9091-9197	5′-GGGGGGACTGGAAGGGATAATTTACTCC-3′

B. Plasmid constructs and riboprobes

Note: pNL4-3 and pNL4-3.Luc.R-E- are abbreviated as pNL4 and pNL4-luc respectively

i) **pNL4-msd**; 1µg pNL4-3 was digested with *ApaI*/*NcoI,* purified by agarose gel electrophoresis, klenow treated and relegated to produce a plasmid designated pNL4-ΔA/N; 50ng was used as a template in PCR mediated site directed mutagenesis. Mutants were identified by sequencing both strands, subcloned into non-template pNL4-ΔA/N using *NdeI* / *EcoNI*, then subcloned into pNL4-3 using *AatII*/*SphI*.

ii) **pNL4-luc.SPA**; a double stranded oligonucleotide containing a *BanI* site and 5′*XhoI*/3′*NgoMIV* linkers was subcloned into *XhoI*/*NgoMIV* digested pNL4-luc (forward oligonucleotide as follows; poly(A) site region underlined; *BanI* site in bold) and confirmed by sequencing both strands:

wild type: 5′-CTCGAG**GGTACC**CCGATCCAATAAAAGATCTTTATT
TTCATTAGATCTGTGTGTTGGTTTTTGTGTGGGCCGGC-3′
mutant: 5′-CTCGAG**GGTACC**CCGATCCGCGGCGAGATCTTTATT
TTCATTAGATCTGTGTGTTGGTTTTTGTGTGGGCCGGC-3′

iii) **pNL4-luc.CMV** was constructed by removing the *FspA1*/*BssHII* fragment of pNL4-luc (containing the HIV-1 5′LTR promoter) and replacing with a *ScaI / BssHII* fragment of CMVmg construct (Ashe et al., 2000). To construct pNL4-luc.DSE, pNL4-luc was digested with *AatII* / *BamHI*, blunt ended by klenow and relegated to form pNL4-ΔA/B; containing the HIV-1 3′LTR only.

iv) **pNL4-luc.DSE** was constructed by removing the U5 region-containing *HindIII* / *NgoMIV* fragment of pNL4-luc..ΔA/B (nt9607-10350) whilst preserving the AAUAAA poly(A) hexamer by klenow-treatment. A *XhoI* / *NcoI* fragment spanning this region was then subcloned into non-template pNL4-luc.

iv) **riboprobes**; pNL4-wt and pNL4-msd were obtained by site directed mutagenesis of the pHmgM1riboprobe (Ashe et al., 1995; see main text).

Figure S1

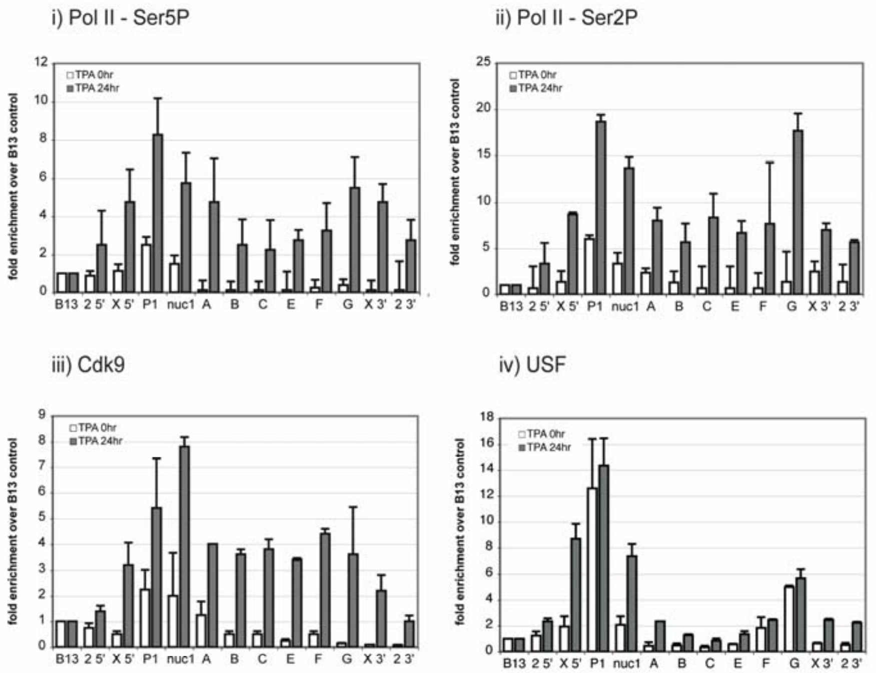

FIGURE S1:
Distribution of Pol II phospho CTD isoforms and associated factors across transcriptionally active provirus

Pol II phospho CTD, Ser5P (i) or Ser2P (ii), CDK9 (iii) and USF (iv) association determined by qChIP assay in control (white bars) and TPA-activated U1 cells after 24hr (grey bars). Immunoprecipitated DNA was analyzed by real-time PCR using the primer sets as described in Figure 1. ChIP signal levels are not comparable between antibodies Note: P1 does not distinguish between the two LTRs. Error bars represent SEM from n=3 samples performed in duplicate for each primer set.

Figure S2

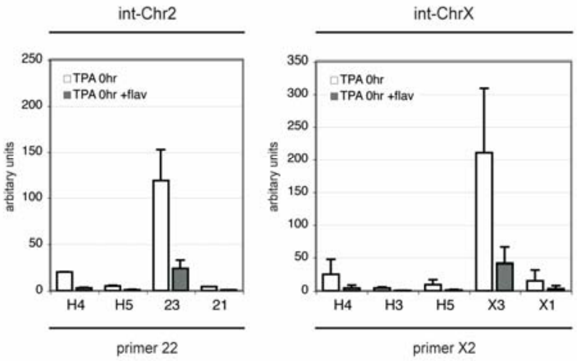

FIGURE S2:
Flavopiridol treatment affects basal U1 HIV-1 gene loop levels

3C analysis of int-Chr2 and int-ChrX before (white bars) and after (grey bars) 5 hours of flavopiridol treatment in the absence of TPA induction. Primer positions and names are as in Figure 2A. Common PCR primers are shown below each graph, with the second primer shown above (as per Figure 5). Error bars represent SEM between n=3 samples performed in duplicate for each primer set from one chromatin preparation.

Figure S3

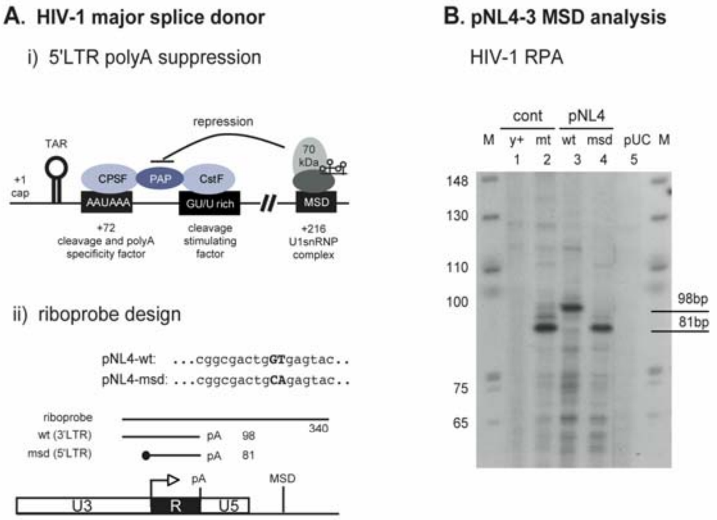

FIGURE S3:
The HIV-1 major splice donor (MSD) mutant causes premature polyadenylation in pNL4-3 transcripts

(A) i) Mechanism of MSD-mediated suppression of the 5' LTR polyA signal (via the U1snRNP complex); interacting RNA processing factors as indicated. ii) LTR-specific riboprobe design and sequence of wild type (wt) and mutant (msd) MSD sequence. (B): RPA shows MSD inactivation in pNL4 (lane 4; mt) leads to premature polyadenylation, A hybrid HIV-1/γ globin gene construct originally employed to show MSD suppression of the 5'LTR poly(A) signal (Ashe et al 1997) was used as a control. RPA was carried out using a riboprobe specific for the HIV-1 LTR poly(A) signals . As expected, pNL4-wt gave an RNAse protected poly(A) band (98nt) showing 3'LTR poly(A) site usage (lane 3) while the MSD mutant either for pNL4-msd or the original mutant plasmid gave a smaller 5'LTR poly(A) site band (81 n; lanes 2 and 4). Specificity of RPA signals was confirmed using pUC18 transfected HeLa cell RNA (lane 5) and yeast RNA (lane 1) controls.

Lanes are as follows; (M) DNA marker; (1) yeast RNA control with wild-type probe (2) pHmgM1 MSD (mt) construct (Ashe et al., 1997), (3) wt; pNL4.3 (4) msd; pNL4.3 with mutant MSD (5) pUC18 control.

Figure S4

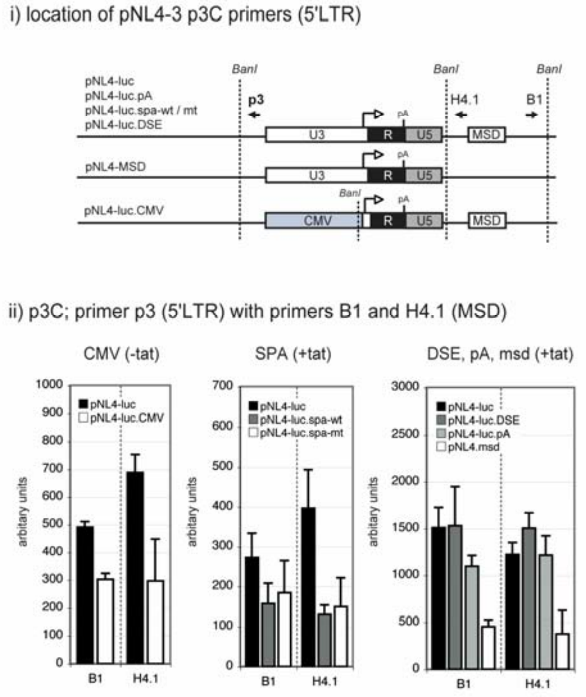

FIGURE S4:

p3C comparison of primer B1 with an alternative reverse facing primer (H4.1)

i) Location of primers used to detect interaction of the HIV-1 5'LTR (primer p3) with the major splice donor region (primer B1). The position of alternative facing MSD p3C primer (H4.1; located at position 672nt; Figure 3) is indicated. Arrows indicate transcription start sites. ii) Real-time q-p3C analysis of comparative loop formation using primer p3 (5'LTR) using pNL4-luc and derivatives (see Figure 6) with (+) or without (-) co-transfected Tat expression vector. Error bars represent SEM between values between two separate chromatin preparations performed in triplicate for each primer set (n=6), except for SPA contructs (n=9).

Figure S5

i)

↓

pNL4-luc: **AAUAAA**GCUUGCCUUGAGUGCUUCAA-GUAGUGUGUGCCCGUCUGUUGUGUGACUCUGGUAACUAGAGAUCCCUCAGACCCUUUUAG

pNL4-luc.DSE: **AAUAAA**GCUCCGGCUGUGCGGGAGAACGGAGUUCUAUUAUGACUCAAAUCAGUCUCCCCAAGCAUUCGGGGAUCAGAGUUUUUAAGG

pNL4-luc.SPA: **AAUAAA**GAUC_UUUAUUUUCAUU-AGAUCUGUGUGUUGGUUUUUGUGUGGGCCGGCUGUGCGGGAGAACGGAGUUCUAUUAUGACUC

ii)

pNL4-luc.pA: CTGGAAGGGCTAATTCACTCCCAAAGAAGACAAGATATCCTTGATCTGTGGATCTACCACACACAAGGCTACTTCCCTGATTGGCAG
AACTACACACCAGGGCCAGGGGTCAGATATCCACTGACCTTTGGATGGTGCTACAAGCTAGTACCAGTTGAGCCAGATAAGGTAGAA
GAGGCC**AATAAA**GGAGAGAACACCAGCTTGTTACACCCTGTGAGCCTGCATGGAATGGATGACCCTGAGAGAGAAGTGTTAGAGTGG
AGGTTTGACAGCCGCCTAGCATTTCATCACGTGGCCCGAGAGCTGCATCCGGAGTACTTCAAGAACTGCTGACATCGAGCTTGCTAC
AAGGGACTTTCCGCTGGGGACTTTCCAGGGAGGTGTGGCCTGGGCGGGACTGGGGAGTGGCGAGCCCTCAGATGCTGCATATAAGCA
GCTGCTTTTTGCCTGTACTGGGTCTCTCTGGTTAGACCAGATCTGAGCCTGGGAGCTCTCTGGCTAACTAGGGAACCCACTGCTTAA
GCCTC**AACAAA**GCTTGCCTTGAGTGCTTCAAGTAGTGTGTGCCCGTCTGTTGTGTGACTCTGGTAACTAGAGATCCCTCAGACCCTT
TTAGTCAGTGTGGAAAATCTCTAGCA*CCCAGGAGGTAGAGGTTGCAGTGAGCCAAGATCGCGCCACTGCATTCCAGCCTGGGCAAGA*

FIGURE S5:

3′LTR Sequence analysis of pNL4-luc with derivatives pNL4-luc.SPA, pNL4-luc.DSE and pNL4-luc.pA

i) Sequence alignment of pNL4-luc reveals the presence of GU and U rich sequences downstream from the 3' polyA site in the pNL4-luc.DSE mutant. The polyA sequence is in bold with regions of sequence homology in the GU and U regions outlined by Levitt et al., 1989 in black. The potential endonuclease cleavage site is denoted by an arrow. Other GU rich regions are shaded in light grey. ii) The U3 region of HIV-1 3′LTR contains an AAUAAA sequence, which may act as a cryptic poly(A) signal in the pNL4-luc.pA mutant. Poly(A) sites are in bold, with the region mutated in this study (AACAAA) within the U5 region as indicated. The TATA box is underlined, R region in light grey, U5 sequence in black and the flanking plasmid sequence in italics.
